# An audit of breast imaging scoring by radiologists, in cases of proven malignancy

**DOI:** 10.1186/bcr2970

**Published:** 2011-11-04

**Authors:** JR Powell, B Mucci

**Affiliations:** 1 Greater Glasgow NHS Trust, Glasgow, UK

## Introduction

In 2009 the Royal College of Radiologists Breast Group set out a classification system for breast imaging scoring [1]. A score of 4 'suspicious of malignancy' or 5 'highly suspicious of malignancy' should be used 'in most cases' of proven breast cancer. However, there is no universally accepted target for this in the UK. The American BIRADS system suggests a 98% target [2]; however, their scoring system is slightly different.

## Methods

We examined imaging reports (mammography, ultrasound and MRI) of patients diagnosed with a breast malignancy attending the symptomatic breast clinic in 2009. Our aim was to determine an achievable target for correctly scoring breast imaging.

## Results

A total of 203 patients' imaging was examined. One hundred per cent of reports included a score. Ninety per cent of the cancers were scored 4 or 5. In three underscored cases, radiologist opinion was clouded by the fact the patient had had proceeding FNA. Cases of cancer recurrence also proved difficult to correctly score.

## Conclusion

We suggest a target of 90 to 95% of all breast cancers being scored 4 or 5. A 95% target is achievable especially if FNA/biopsy is left until after imaging has been performed.
